# Seminoma Presenting as Renal Mass, Inferior Vena Caval Thrombus, and Regressed Testicular Mass

**DOI:** 10.1155/2015/835962

**Published:** 2015-01-29

**Authors:** Valary T. Raup, Michael H. Johnson, Jonathan R. Weese, Ian S. Hagemann, Stephen D. Marshall, Steven B. Brandes

**Affiliations:** ^1^Division of Urologic Surgery, Department of Surgery, Washington University School of Medicine, Saint Louis, MO, USA; ^2^Division of Anatomic and Molecular Pathology, Department of Pathology and Immunology, Washington University School of Medicine, Saint Louis, MO, USA

## Abstract

Testicular cancer is the most common malignancy of men aged 15–40. Metastatic spread classically begins with involvement of the retroperitoneal lymph nodes, with metastases to the liver, lung, bone, and brain representing advancing disease. Treatment is based on pathologic analysis of the excised testicle and presence of elevated tumor markers. We report a case of a 34-year-old male presenting with back pain who was found to have a right renal mass with tumor extension into the inferior vena cava. Subsequent biopsy was consistent with seminoma. We review this rare case and discuss the literature regarding its diagnosis and management.

## 1. Introduction

Testicular cancers are the most common malignancy for men aged 15–40 [1]. Of these, seminoma accounts for approximately 45% of tumors, and patients typically present with a testicular mass. When metastatic, the primary landing sites are the retroperitoneal lymph nodes. Metastases to the liver, lung, bone, and brain represent more advanced disease. Treatment is based on pathologic analysis of the excised testicle, evidence of metastasis to the retroperitoneal lymph nodes or elsewhere, and the presence or absence of elevated tumor markers. Treatment for seminoma may involve systemic chemotherapy, radiation, and/or extirpative surgery.

## 2. Case Presentation

**Figure 1:**
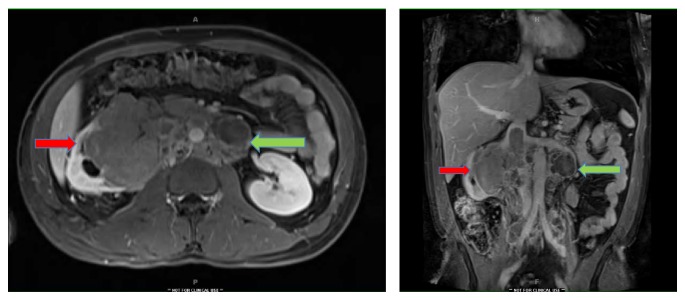
MRI showing right renal mass with IVC thrombus (red arrows) and retroperitoneal lymphadenopathy (green arrows).

A 34-year-old Latino man was referred to clinic with a diagnosis of a renal mass with inferior vena caval thrombus. This was initially found after he reported a one-month history of back pain. He denied any hematuria, pain, weight loss, lower extremity edema, and his physical exam was unremarkable. An MRI showed an 8 cm renal mass with tumor thrombus extending into the IVC, below the diaphragm (Figure 1).

**Figure 2:**
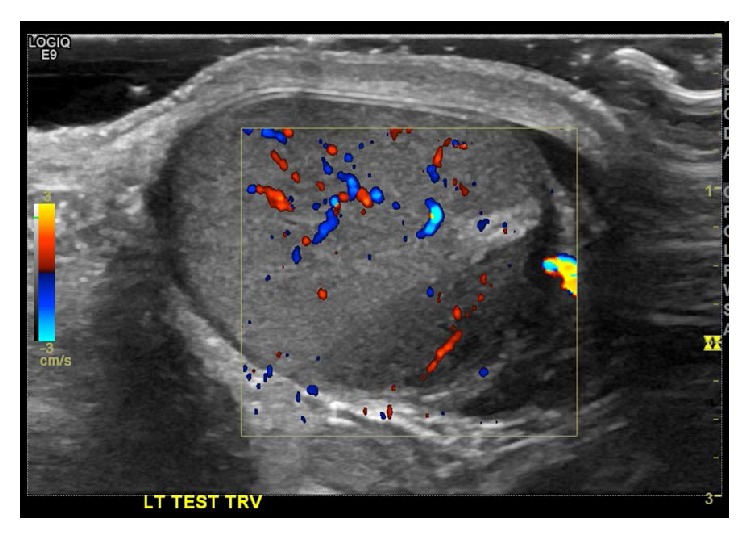
Testicular ultrasound showing hypoechoic lesion in left testis.

Additionally, he had bilateral retroperitoneal lymphadenopathy and a left supraclavicular mass. His LDH was elevated at 408 and his AFP and HCG were both within normal limits. Given his age, elevated tumor markers, and extensive adenopathy concerning for testicular cancer, he underwent a scrotal ultrasound, which demonstrated a hypoechoic left testicular lesion (Figure 2).

**Figure 3:**
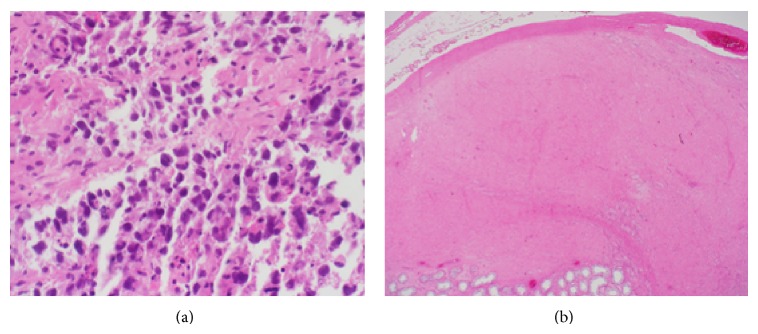
(a) Renal biopsy showing classic seminoma histology. H&E, original magnification 200x. (b) Testis showing fibrosis consistent with regressed seminoma. H&E, original magnification 20x.

The patient initially underwent a biopsy of the supraclavicular lymph node, but the histology showed only necrosis and was noncontributory. He then underwent a biopsy of the renal mass as well as a subsequent left inguinal orchiectomy. Histologically, the renal biopsy supported the diagnosis of metastatic seminoma, showing homogeneous, medium-sized tumor cells that were loosely organized in nests, surrounded by fibrous stroma with a lymphocytic infiltrate, with no renal cortex or medulla seen. No granulomas were identified. Immunohistochemical staining showed that the tumor cells were positive for SALL4, vimentin, c-Kit, and OCT4 and negative for CD30, desmin, SMA, and cytokeratins AE1/AE3. The orchiectomy specimen grossly showed unremarkable brown spongy parenchyma, with an ill-defined, tan-yellow shiny area 1.3 cm in greatest dimension and confined to the testes. Histologically, the testis excision showed marked fibrosis consistent with regressed seminoma. No viable tumor was found (Figure 3).

**Figure 4:**
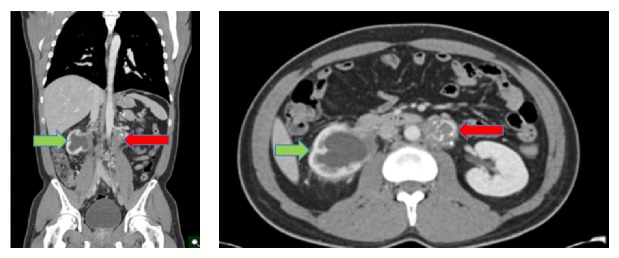
CT scan obtained 8 months after presentation status postchemotherapy showing reduction in the soft tissue stranding and thickening of the right kidney (green arrows) as well as stability of the retroperitoneal lymph nodes (red arrows).

The patient was subsequently referred to medical oncology for chemotherapy and further treatment. An IVC filter was placed cephalad to the thrombus and he was started on bleomycin, etoposide, and cisplatin (BEP) systemic chemotherapy with side effects of watery diarrhea and fatigue. During treatment with BEP, he was admitted to the hospital due to development of neutropenic fever, severe mouth pain, and genital lesions consistent with HSV infection. After 3 cycles of BEP, he developed pulmonary toxicity and bleomycin was discontinued for the fourth cycle. He underwent a CT scan six months after presentation showing substantial interval decrease in the size of bilateral retroperitoneal, pelvic, and left lower cervical lymphadenopathy. Marked interval decrease in the size of the IVC thrombus was also noted. These changes were thought to be consistent with necrosis secondary to chemotherapeutic effect on the lymphadenopathy. New scattered ground glass airspace opacities in the bilateral mid- and lower lungs were also seen, consistent with his bleomycin induced pulmonary toxicity. The patient received two additional cycles of EP, after which the patient underwent a PET scan (now seven months postpresentation). This PET scan showed mild retroperitoneal enhancement consistent with resolving necrosis. Repeat CT scan obtained eight months after presentation and treatment showed no change in the partially calcified lymph nodes in the retroperitoneum and an unchanged stable eccentric thrombus of the IVC (Figure 4). There was no evidence of metastasis or recurrence. The patient will be followed radiographically for recurrence every three to six months for five years as per NCCN Guidelines for stage III intermediate-risk seminoma. If the patient is found to have a recurrence, his multidisciplinary team will evaluate the need for additional chemotherapy, radiation, or retroperitoneal lymph node dissection.

## 3. Discussion

Testicular cancer is the most common cancer in males between the ages of 15 and 40 [1]. Across all ages, there will be an estimated 8,820 new cases and 380 deaths in 2014 [2]. 95% of these are germ cell tumors, including seminoma and nonseminomatous germ cell tumors (NSGCT). Metastatic spread of these tumors typically occurs via retroperitoneal lymphatics, and most common sites include retroperitoneal lymph nodes, lungs, liver, brain, and bone. Retroperitoneal tumors diagnosed as seminoma are virtually always metastases and will usually have evidence of a corresponding primary intratesticular malignancy. However, extensive fibrosis can make the pathologic diagnosis difficult, and complete regression of the tumor can occasionally be seen [3].

Grossly, intratesticular seminoma presents as a cream or tan-colored nodular mass. Microscopically, confluent sheets of cells are seen with clear or eosinophilic cytoplasm. The sheets are classically divided by fibrous bands containing a prominent lymphocytic infiltrate. In both primary tumors and metastases, granulomas are a classic feature. Syncytiotrophoblast can be seen and should not be overinterpreted as evidence of choriocarcinoma. Seminomas typically stain positively for placental alkaline phosphatase (PLAP), CD117/c-Kit, OCT3/4, SALL4, vimentin, and D2-40. In contrast, they are negative for CD30, desmin, SMA, and pan-cytokeratin. These immunostains are useful in differentiating subtypes of germ cell tumors and identifying minor components, as mixed germ cell tumors are common.

Ultrasonography remains the standard imaging modality for testicular malignancy, and CT with oral and intravenous contrast is preferred for diagnosis of retroperitoneal lymphadenopathy. While this study focuses predominately on seminomatous testicular tumors, the NCCN recommends postdiagnostic abdomen and pelvis CT scan, with or without chest imaging, for both seminoma and NSGCT. Additionally, bone scan or brain MRI should be performed as clinically indicated [4]. Tumor markers (LDH, HCG, and AFP) should be collected postorchiectomy for cancer staging. Following orchiectomy, seminoma can be further treated with radiation or medical therapy. NCCN guidelines recommend etoposide and cisplatin, with or without bleomycin, as the initial chemotherapy for metastatic disease. Anticoagulation is commonly initiated concurrently with chemotherapy, and retroperitoneal lymph node dissection with/without further surgical exploration is often required for residual disease.

Renal cell carcinoma frequently metastasizes to the lumen of the IVC, but seminoma can also mimic this picture. Case reports exist of IVC involvement on initial presentation, although involvement is more typically found after diagnosis [5]. In a 20-year retrospective review of testicular cancer patients at a single institution, the incidence of intraluminal thrombus at postchemotherapy retroperitoneal lymph node dissection was found to be 5.8% [6]. The study identified 89 postchemotherapy patients with 98 intraluminal thrombi (72 IVC, 20 renal vein, and 1 aorta), and active malignancy was found in approximately 45%. Of the patients with intraluminal thrombus, one-half eventually required vena cavectomy for curative treatment.

## 4. Conclusion

Testicular cancer is a common malignancy for males aged 15–40. Initial presentation involving an IVC tumor thrombus is rare, especially in the setting of regressed testicular tumor. Treatment should follow the standard guidelines of testicular and retroperitoneal imaging, orchiectomy, and chemotherapy. Pulmonary embolus precautions should be taken, and patients should be counseled that retroperitoneal lymph node dissection with vascular reconstruction may be necessary.

## Abbreviations

BEP: Bleomycin, etoposide, and cisplatin
EP: Etoposide and cisplatin
H&E: Hematoxylin and eosin
IVC: Inferior vena cava
NCCN: National Comprehensive Cancer Network
NSGCT: Nonseminomatous germ cell tumor.
